# Mixed- and multi-relative biological effectiveness model simultaneous optimization in carbon ion radiotherapy: A proof-of-concept

**DOI:** 10.1016/j.phro.2024.100679

**Published:** 2024-11-20

**Authors:** Aaron Paul Osburg, Peter Lysakovski, Giuseppe Magro, Semi Harrabi, Thomas Haberer, Amir Abdollahi, Jürgen Debus, Thomas Tessonnier, Andrea Mairani

**Affiliations:** a Faculty of Physics and Astronomy, Heidelberg University, Im Neuenheimer Feld 226, 69120 Heidelberg, Germany; b Heidelberg Ion-Beam Therapy Center (HIT), Im Neuenheimer Feld 450, 69120 Heidelberg, Germany; c Heidelberg Institute of Radiation Oncology (HIRO), National Center for Radiation Oncology (NCRO), Heidelberg University Hospital (UKHD), Heidelberg Faculty of Medicine (MFHD), Heidelberg, Germany; d Clinical Cooperation Unit Translational Radiation Oncology, German Cancer Consortium (DKTK) Core-Center Heidelberg, National Center for Tumor Diseases (NCT), Heidelberg University Hospital (UKHD) and German Cancer Research Center (DKFZ), Heidelberg, Germany; e Division of Molecular and Translational Radiation Oncology, Heidelberg Faculty of Medicine (MFHD) and Department of Radiation Oncology, Heidelberg University Hospital (UKHD), Heidelberg, Germany; f Medical Physics Unit, National Center for Oncological Hadrontherapy (CNAO), Pavia, Italy

## Abstract

**Background and purpose:**

In carbon ion radiotherapy (CIRT), different relative biological effectiveness (RBE) models have been used for calculating RBE-weighted dose (D_RBE_). Conversion between current RBE predictions and introduction of novel approaches remains a challenging task. Our aim is to introduce a framework considering multiple RBE models simultaneously during CIRT plan optimization, easing the translation between D_RBE_ prescriptions.

**Materials and methods:**

An in-house developed Monte Carlo treatment planning system was extended to incorporate the local effect model version I (LEM-I), the modified microdosimetric kinetic model (mMKM) and the MKM-derived Japanese biological model (NIRS-MKM). Four clinical cases (two head-and-neck and two prostate patients), initially optimized with LEM-I for both targets and organs at risk (OARs), underwent two further optimizations: to fulfill mMKM/NIRS-MKM-based target prescriptions (mixed-RBE approach) or to simultaneously consider two biological models within the target regions (multi-RBE approach). Both approaches retained LEM-I-derived dose constraints for OARs.

**Results:**

The developed optimization strategies have been successfully applied, fulfilling all the clinical criteria for the applied RBE models. One of the RBE models showed unfavorable dose distribution when not explicitly considered in the optimization, while multi-RBE model optimization allowed meeting dose objectives for the selected OARs for both models simultaneously.

**Conclusions:**

The introduced optimization approaches allow for mixed- or multi-RBE optimization in CIRT through the selection of RBE models independently for each region of interest. This capability addresses challenges of adhering to multiple RBE frameworks and proposes an advanced solution for tailored patient treatment plans.

## 1 Introduction

The determination of optimal dose prescription in carbon-ion radiotherapy (CIRT) presents an ongoing area of investigation. This challenge arises since no definitive consensus exists on the optimal biological framework for predicting relative biological effectiveness (RBE). Historically, European and select Asian facilities have adhered to the local effect model version I (LEM-I) [1], while Japanese institutions have adopted either the semi-phenomenological mixed-beam-model [2] or the modified microdosimetric kinetic model (mMKM), incorporating customized input parameters to align with clinical outcomes at NIRS [3]. Both LEM-I and mMKM are designed to predict RBE distributions (doses in Gy(RBE)_LEM-I_ and Gy(RBE)_mMKM_), albeit grounded in disparate theoretical constructs and assumptions [4]. Both models have variable inputs and free parameters, customized for clinical applications and based on specific cell lines. In terms of RBE prediction, LEM-I is known to overestimate the effective dose, particularly in the entrance/low-linear energy transfer (LET) region. Conversely, mMKM has shown greater accuracy *in-vitro*, *in-vivo* and in patients [5], [6]. LEM-I and the NIRS approach (NIRS-MKM, the Inaniwa's mMKM-derived approach– Gy(RBE)_JP_ –consistent with prior clinical experience [3]) incorporate different dose dependences further complicating comparisons. This disparity leads to non-equivalent biological effectiveness even when prescribed doses in Gy(RBE) appear numerically identical. Consequently, effective dose transfer and clinical outcome comparisons across centers utilizing different models are highly challenging and can influence clinical decisions [7], [8].

European centers have adopted Japanese CIRT treatment protocols to benefit from NIRS's clinical experience [9]. However, a major challenge lies in establishing dose constraints for organs at risk (OARs). While prescription doses and fractionation schemes have been adapted from NIRS due to their extensive experience, some OARs dose constraints were initially adopted conservatively, i.e. not adjusted to account for RBE dependence. This cautious approach was taken to mitigate uncertainties related to differences in RBE models, beam delivery methods and dose optimization processes. This conservative strategy, implemented at the beginning of clinical activity to minimize the risk of unexpected side effects, could have potentially limited target coverage and compromised treatment efficacy. Recent analyses have addressed this issue by integrating detailed RBE model translation and assessing treatment toxicity. They drew upon long-term clinical follow-up data and predictions of normal tissue complications derived from other CIRT centers [10], [11], [12], [13], [14], [15], [16]. Highlighting the uncertainty in RBE models [5], European centers are increasingly adopting a hybrid approach for CIRT, aiming to bridge the gap between historical practice and the potentially superior mMKM-based biological dose prediction. This mostly involves optimizing with LEM-I and recalculating with mMKM/NIRS-MKM, repetitively, by manual iterations until reaching satisfactory alignment with both models’ constraints [14]. However, this process is time-consuming, inefficient, and does not ensure favorable outcomes due to the multitude of available degrees of freedom (number of beams, beams’ orientation and range, etc.), set manually by the planner. However, the specific methodology for this technique remains unclear and case-dependent, hindering broader application. Therefore, the hybrid approach may not be suitable for all patients and, sometimes, the benefits may not outweigh the additional complexity and costs. Additionally, it is crucial to take into account practical clinical implications, such as ensuring that established constraints for OARs are still met when transitioning to a new biological model. Our goal is to merge the clinically used LEM-I and mMKM/NIRS-MKM into a single optimization framework. This integration aims to minimize differences in dose recalculations between two models, ensuring seamless transition and allowing, for example, to continue using constraints established through LEM-I-based experiences for European centers. To support this, the work explores new functionalities of MonteRay, a previously validated fast Monte Carlo (MC) code [17]. A crucial achievement of this work is the development and integration of a fluence-optimization module that is interfaced with the dose calculation module enabling plan optimization across a spectrum of RBE models.

## 2 Materials and methods

### 2.1 Monte Carlo treatment planning system based on MonteRay

In this study, three radiobiological models, namely the LEM-I, the mMKM, and its variant– the NIRS-MKM –were employed. The distinction between the latter and mMKM lies solely in the initialization parameters, reference radiation, and a scaling factor, while maintaining consistency in its microdosimetric implementation (supplementary material (SM)). As a result, direct dosimetric comparison between LEM-I and mMKM was possible for the selected adenoid cystic carcinoma (ACC) cases, given that input tables were generated referencing to the same (α/β)_x_ and biological endpoint for both LEM-I and mMKM. For the prostate cancer (PCa) cases, a scaling of the nominal prescription dose defined in LEM-I was instead necessary to ensure a fair comparison with NIRS-MKM. Following [8], [9], LEM-I prescription dose per fraction– defined at the median dose –has been derived as *D*_50%_ = 1.153 [Gy(RBE)_LEM-I_/Gy(RBE)_JP_] × 3.60 [Gy(RBE)_JP_] = 4.15 Gy(RBE)_LEM-I_.

Within this work, a novel GPU-accelerated fluence-optimizer has been developed (SM). The novel optimization framework utilizes MonteRay’s capability to perform biological dose calculations using LEM-I, mMKM and NIRS-MKM, facilitating the incorporation of dose-influence data from multiple RBE models into the optimization process. To the best of our knowledge, this is the first optimization framework capable of integrating multi-RBE model dose-influence data for carbon ions, while offering a wide range of clinically established cost-functions and constraint types.

The integration of LEM-I, mMKM and NIRS-MKM was exploited to facilitate multi-RBE optimization within the fluence-optimization module. To demonstrate its capability, optimization was conducted to adhere simultaneously to dose constraints in both mMKM/NIRS-MKM and LEM-I, independently selecting the most suitable model for each region of interest (ROI) and/or defining cost-function terms based on both models within the same ROI. Finally, the core objective was the usage of the mMKM/NIRS-MKM for target irradiation, while adhering to established clinical constraints derived from the LEM-I for OARs.

### 2.2 Clinical cases and optimization strategies

This study provides a proof-of-concept illustration of simultaneous integration of multiple RBE models. It was conducted in accordance with institutional guidelines and the Declaration of Helsinki (2024). Patient confidentiality was safeguarded by anonymizing data, and written informed consent for research use was obtained from each patient. We restricted our investigation to two distinct anatomical sites (two cases each): ACC of the head-and-neck region and PCa of the pelvic region. All cases were initially optimized using the established LEM-I model as summarized in the SM. The optimization adhered to clinically approved objectives and constraints following current patient protocols at HIT or at CNAO. The second case of each anatomical district (ACC-2, PCa-2) is presented in SM.

In terms of target goal, the requirement was for at least 95% of the prescription dose to cover 95% of the clinical target volumes (CTVs), irrespective of the radiobiological model adopted. A summary of the selected test cases is given in Table 1 (and SM1), in terms of prescription dose, CTV size, and fields’ arrangement. The three optimization scenarios are described in the following:

A. the plan underwent conventional optimization to meet clinical dose-objectives based on the LEM-I, employing cost-function and constraints exclusively derived from LEM-I (ACC-1.A, PCa-1.A);
B. subsequently, the optimization focused on achieving identical dose-objectives for the target volumes but in terms of mMKM(ACC)/NIRS-MKM(PCa) dose, while maintaining dose constraints for OARs based on LEM-I (ACC-1.B, PCa-1.B);
C. finally, the optimization objective shifted towards fulfilling dose-objectives for the target volumes based on both LEM-I and mMKM(ACC)/NIRS-MKM(PCa) model, keeping dose constraints for OARs based on LEM-I (ACC-1.C, PCa-1.C).

**Table 1:** Summary of the treatment plans’ features for the selected test cases: adenoid cystic carcinoma (ACC) for the head-and-neck region, and prostate cancer (PCa) for the pelvic region.

Case name-number	LEM-I Prescription dose [Gy(RBE)_LEM-I_]	mMKM/NIRS-MKM Prescription dose [Gy (RBE)_mMKM/JP_]	LEM-I Dose per fraction [Gy (RBE)_LEM-I_]	mMKM/NIRS-MKM Dose per fraction [Gy (RBE)_mMKM/JP_]	Number of fractions	CTV size[cc]	Fields’ arrangement
ACC-1	60.0	60.0*	3.00	3.00*	20	170.0	3 non-coplanar beams
PCa-1	66.4	57.6**	4.15	3.60**	16	53.3	2 opposite beams

* Gy(RBE)_mMKM_ (mMKM).

** Gy(RBE)_JP_ (NIRS-MKM).

The rationale behind the choice of models lies on the specific clinical indications. For ACC target volumes, which may be treated sequentially with photon plans followed by a carbon boost, the mMKM model— developed to predict the photon-equivalent response of ions —was chosen as the reference. In the case of PCa target volumes, treated exclusively with carbon ions, a model that replicates the Japanese experience was employed. For the OARs, the LEM-I model was consistently used as the reference, as clinicians' experience with toxicity at our institutions is based on this model.

### 2.3 Result evaluation

Following optimization based on the described scenarios, each plan underwent recalculation utilizing the selected models. OARs were solely tested against LEM-I-derived objectives. Dose-volume-histogram (DVH) metrics, such as *D*_98%_ or *D*_2cc_, were adopted as numerical surrogates for target coverage and overdosage. The median CTV dose (*D*_50%_) was used to monitor the LEM-I to NIRS-MKM scaling factor, to assess outcome’s compatibility with the [8], [9]’s approach. CTV average dose was employed to evaluate the overall dose. The homogeneity index (HI [%] = (*D*_2%_ − *D*_98%_)/*D*_50%_) [18] was calculated and deemed acceptable when below ∼17% [19].

## 3 Results

**Fig. 1:**
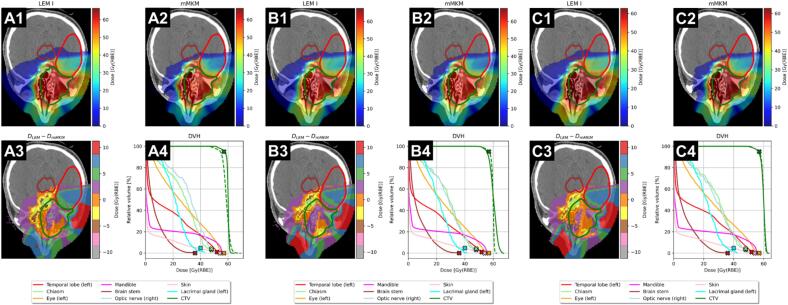
ACC-1 case. Panels A) LEM-I-derived clinical goals for both OARs and targets; panels B) LEM-I-derived clinical goals for OARs and mMKM-derived clinical goals for targets; panels C) LEM-I-derived clinical goals for OARs and simultaneous LEM-I/mMKM-derived clinical goals for targets. Subfigures 1, 2, 3 and 4 depict resulting LEM-I, mMKM, dose difference distributions and DVHs. DVHs for LEM-I and mMKM are displayed as solid and dashed lines, respectively. The cross-marks represent selected LEM-I dose-objectives from Table SM3.

**Fig. 2:**
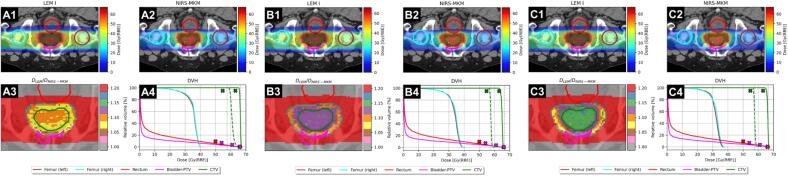
PCa-1 case. Panels A) LEM-I-derived clinical goals for both OARs and targets; panels B) LEM-I-derived clinical goals for OARs and NIRS-MKM-derived clinical goals for targets; panels C) LEM-I-derived clinical goals for OARs and simultaneous LEM-I/NIRS-MKM-derived clinical goals for targets. Subfigures 1, 2, 3 and 4 depict resulting LEM-I, NIRS-MKM, dose ratio distributions and DVHs. DVHs for LEM-I and NIRS-MKM are displayed as solid and dashed lines, respectively. The cross-marks represent the LEM-I dose-objectives from Table SM5. Vertical dotted lines in subfigures 4 represent nominal dose prescription at *D*_50%_, separately for the two models.

Fig. 1, Fig. 2 (and SM1, SM2) present the calculation results. Each figure is subdivided into panels (A, B and C), corresponding to the respective scenario. Within each panel subgroup, a numbering system guides the interpretation:

1. dose distribution recalculated with LEM-I;
2. dose distribution recalculated with the mMKM for ACC or NIRS-MKM for PCa;
3. for ACC, difference map highlighting discrepancies between LEM-I and mMKM dose distribution; for PCa, ratio (LEM-I/NIRS-MKM) depicting dose variations between the two models;
4. resulting DVHs for OARs and for target volumes for the two models.

Tables 2 and SM2 detail the dose-objectives achieved for the test cases under each scenario. Specific numerical values for the CTV are provided using both LEM-I and mMKM for ACC or NIRS-MKM for PCa. Additionally, the number of OARs selected for each optimization problem is reported, with the corresponding optimization outcome based on LEM-I-derived dose-goals. Explicit numerical values for these latter have been listed in Tables SM3 to SM6.

**Table 2:** Clinical goals for the selected test cases (ACC-1 and PCa-1) for each optimization scenario. Explicit numeric outcomes referring to the clinical target volume (CTV) are reported in terms of LEM-I and recalculated mMKM (ACC) or NIRS-MKM (PCa) biological doses. Additionally, the number of regions of interest (ROIs) achieving the desired dose-objectives referring to the LEM-I for all organs at risk (OARs) is presented. The numerical findings are detailed in the referenced table of the supplementary material. A cross-mark denoting non-adherence (✕) to the dosimetric objectives for easier interpretation has been used.

Case name-number	ROI	Clinical goal [Gy(RBE)_LEM-I_]	ACC-1.A, PCa-1.A		ACC-1.B, PCa-1.B		ACC-1.C, PCa-1.C	
			LEM-I Value [%]	mMKM/NIRS-MKM Value [%]	LEM-I Value [%]	mMKM/NIRS-MKM Value [%]	LEM-I Value [%]	mMKM/NIRS-MKM Value [%]
*ACC-1*	CTV	*V*_57.0_ ≥ 95.0 %	95.5	78.8 (✕)	88.7 (✕)	95.0	93.3 (✕)	95.0
	OARs (14)	*(see*Table SM3 in supplementary material*)*	all fulfilled	−	all fulfilled	−	all fulfilled	−
PCa-1	CTV	*V*_54.72/63.08_* ≥ 95.0 %	100.0	100.0	99.5	100.0	100.0	100.0
	OARs (5)	*(see*Table SM5 in supplementary material*)*	all fulfilled	−	all fulfilled	−	all fulfilled	−

* *V*_LEM-I/NIRS-MKM_: subscripted dose values correspond to 95% of the prescription dose as according to the LEM-I and NIRS-MKM.

### 3.1 LEM-I biological dose optimization (scenario A)

The optimizations based on LEM-I reproduced treatment plans approved for clinical use.

**ACC-1.A**. *D*_50%_ deviated from the prescribed dose by less than 0.1%, D_98%_ was 90.3% of the prescribed dose, while *D*_2cc_ reached 102.7%, indicating successful attainment of prescription levels and target coverage without any undesired hotspots. The HI = 11.9% further confirmed the relatively high degree of homogeneity of the LEM-I optimized dose. Conversely, the recalculated mMKM exhibited poor performance, with significantly CTV lower *D*_50%_ (−2.1%), *D*_98%_ (−16.0%) and failure to meet clinical goals for target coverage. Additionally, the CTV’s DVH displayed high-dose tails exceeding safe limits relative to the prescribed dose (*D*_2cc_ = 106.9%), resulting in a high HI value (22.2%). The dose difference map showed significant regions where the discrepancy between the two competing models was high, particularly at the distal part of the target with respect to the beams’ entrance.

**PCa-1.A**. Given the limited anatomical heterogeneity of the pelvic region, the dose distribution achieved with LEM-I on the CTV appeared extremely homogeneous (HI = 2.1%), with no evidence of under- or overdosage (*D*_98%_ = 98.8%, *D*_2cc_ = 100.7%) compared to the prescription dose. Examining the DVH of the recalculation using the NIRS-MKM, overall, there was a slight loss of dose homogeneity (HI = 7.8%), hotspots were observed (*D*_2cc_ = 110.0%) and, most importantly, the entire target area appeared significantly overdosed (average dose of ~60.9 Gy(RBE)_JP_ versus the prescribed value of 57.6 Gy(RBE)_JP_). The ratio between median doses of the CTV when optimizing in LEM-I and recalculating in NIRS-MKM (*F*_scal_) was markedly lower (1.09) than what predicted by the theoretical model (1.153) [8], [9].

### 3.2 Mixed-RBE model optimization (scenario B)

**ACC-1.B**. LEM-I dose constraints for OARs were met, while simultaneously enhancing the CTV coverage with the mMKM biological dose (*D*_98%_ = 90.3%), without registering hotspots (*D*_2cc_ = 102.3%). The HI of 11.8% further confirmed the homogeneity achieved by the mMKM-optimized plan on the CTV. Clinical goals for target coverage failed to be met with LEM-I. In the LEM-I plan, *D*_98%_ decreased to 88.7%, while the *D*_2cc_ value notably increased to 109.7%, resulting in higher HI (20.2%). Furthermore, the same pattern as scenario A was observed in this case, concerning dose difference maps.

**PCa-1.B**. Both dose distributions exhibited comparable HI (HI_LEM-I_ = 3.7%, HI_NIRS-MKM_ = 3.5%). However, the LEM-I distribution consistently underestimated the dose level when compared to the rescaled value as according to the theoretical model (CTV *D*_50%_ was 2.1% less than the LEM-I prescription dose level). Conversely, the dose distribution in NIRS-MKM improved strongly compared to PCa-1.A, fully meeting the prescription homogeneously within the target (*D*_50%_ = 100.0%, *D*_98%_ = 98.3% and *D*_2cc_ = 101.5% of the NIRS-MKM prescription dose). The resulting *F*_scal_ was 1.128.

### 3.3 Multi-RBE model optimization (scenario C)

**ACC-1.C**. All dose constraints derived from LEM-I have been adhered to. Similarly, the required dose-objectives for the CTV were met (mMKM) or nearly met (LEM-I, *V*_95%_ ≥ 95% violated by 1.7%). Neither model, when utilized concurrently for optimization, exhibited notable underdosage or hotspots within the target area or in proximity of the OARs (*D*_98%_ was 10.4% and 10.5% less than the prescribed dose for LEM-I and mMKM, respectively, while *D*_2cc_ exceeded by 3.7% and 3.0% the prescribed dose for LEM-I and mMKM, respectively). The comparable HI (HI_LEM-I_ = 13.8%, HI_mMKM_ = 13.2%), and median (*D*_50%_ = 99.9% and 100.0% for LEM-I and mMKM) dose levels confirmed the similarity of the two competing dose distributions. Differences between the two biological doses appeared less pronounced.

**PCa-1.C**. HI was ~3%, median CTV doses differed by less than 0.5% from the nominal prescription value, *D*_98%_ ≥ 98% and *D*_2cc_ < 101.6% of the prescription level for both LEM-I and NIRS-MKM. The dose-objectives derived from LEM-I for OARs were fully achieved, as well as the coverage of the CTV for both models. Upon examining the ratio map, the theoretical scaling factor of ~1.15 was uniformly attained.

## 4 Discussion

**Scenario A.** The initial optimization, exclusively employing LEM-I cost-function parameters for both the target volumes and OARs, resulted in plans that successfully achieved the LEM-I dose-objectives. Conversely, the mMKM dose distributions (ACC cases) were notably unsatisfactory since the optimization process did not take into account explicitly any mMKM dose constraints. For the PCa cases instead, despite a minor increase in HI_NIRS-MKM_, the value remained within a clinically acceptable range [19] (found values < 8%). These aligned with [20].

**Scenario B.** The novel methodology was effectively showcased in the second scenario, wherein the optimization of the target dose distribution relied on mMKM/NIRS-MKM dose predictions, while adhering to LEM-I dose constraints for the OARs. This resulted in plans that upheld the LEM-I dose constraints in the OARs and achieved the mMKM/NIRS-MKM dose-objectives for CTV. Given the tissue heterogeneity of the two ACC cases, the number and the vicinity of the involved OARs and the substantial fluence modulation of the irradiation beams, LEM-I distributions resulted to be clinically unacceptable. For the two PCa cases, despite a marginally higher dose level than predicted by the models by [8], [9], the homogeneity of the CTV dose was maintained through the recalculated LEM-I dose, in line with [20].

**Scenario C.** To prevent significant overdosage or underdosage within the target volumes when using separately LEM-I or mMKM/NIRS-MKM, both models were concurrently considered during the third optimization scenario. Multi-RBE model optimization allowed for meeting, or nearly meeting, dose-objectives for both LEM-I and mMKM/NIRS-MKM simultaneously, without significantly compromising the quality of DVHs for either model, while still meeting the LEM-I dose constraints for the OARs.

The successful reoptimization of patient cases highlighted the practical viability of the new MonteRay dose optimization module in clinical settings, affirming the feasibility and advantages of employing a multi-RBE approach. This approach could benefit from considering a greater number of beams, offering the optimizer increased flexibility in achieving the desired dose distribution across competing models. ACC patient plans offered, in that sense, a practical example: in the ACC-2 case (SM), it was demonstrated that two ipsilateral beams were adequate for effective multi-RBE model optimization. However, the ACC-1 case supports the intuitive expectation that utilizing three non-coplanar beams increases the set of feasible dose distributions, which may enhance the similarity of target DVHs and reduce voxelwise dose discrepancies. While this additional degree of freedom is likely to lead to improved dose distributions, it is not guaranteed in every scenario.

In general, the multi-RBE model optimization strategy allowed lowering the discrepancies between the two models’ predictions compared to the single- or mixed-RBE optimizations, which only employed one model in the target volumes, where HI values reaching o exceeding 20% were noted.

PCa cases, and all prescriptions derived from NIRS-MKM to LEM-I conversion, deserve further consideration. As emphasized by [8], [9], the scaling factor is based on median dose ratios to the target, but also relies on dose distributions optimized in water for simple geometries. While this approach has been confirmed for real anatomies [9], it inherently biases the mixed-RBE approach to match NIRS-MKM and LEM-I median doses.

Albeit with different goals, the multi- and mixed-RBE concretely address the clinical need to translate constraints and dose prescriptions from one radiobiological model to another. However, further investigation across different clinical indications is needed to confirm the generalizability of these findings and to evaluate whether the same reductions in discrepancies between models can be consistently achieved.

Optimizing with a multi-RBE model can be particularly useful for a transitioning center facing complex planning challenges. For example, a strategy may involve initial optimization using LEM-I followed by iterative recalculations in mMKM/NIRS-MKM. In such cases, additional degrees of freedom, like increasing the number of fields, may be introduced until the final dose distribution meets the constraints for both models [14].

However, this approach involves trade-offs, as optimizing for one model may result in suboptimal dose distributions in the other. Adjusting parameters such as incorporating LET-specific terms in the cost-function or modifying the beam's radiological depth to enhance high LET release in targeted regions could mitigate these issues [21], [22], but may also lead to more complex physical dose distributions, potentially reducing the clinical feasibility of the plan without careful evaluation.

Yet, through multi-RBE optimization techniques, it is possible to achieve significant improvements in one criterion (e.g., mMKM) without heavily compromising the first (e.g., LEM). Using only LEM or mMKM tends to produce poor distributions in the other model, but incorporating both models allows for notable improvements in one criterion with only minimal impact on the other. While this adds complexity, it can ultimately lead to more balanced and clinically feasible dose distributions.

A mixed-RBE approach would therefore further ease the challenge of managing cases where LEM constraints are applied cautiously, as meeting these constraints may not ensure compliance with mMKM/NIRS-MKM recommendations without risking target coverage. With this approach, LEM constraints can still be adhered to as a precautionary measure [11], [12], but target coverage can be more reliably managed using the mMKM/NIRS-MKM model, resulting in a clinically acceptable plan.

This procedure could support the transition to new radiobiological models in clinical protocols by ensuring consistency in monitoring adverse effects (with OAR constraints in LEM) and enabling direct comparison with the extensive Japanese experience on local control (due to target optimization in NIRS-MKM).

## CRediT authorship contribution statement

**Aaron Paul Osburg:** Data curation, Formal analysis, Investigation, Methodology, Software, Validation, Visualization, Writing – original draft, Writing – review & editing. **Peter Lysakovski:** Investigation, Methodology, Software, Validation, Visualization, Writing – original draft, Writing – review & editing. **Giuseppe Magro:** Conceptualization, Validation, Writing – original draft, Writing – review & editing. **Semi Harrabi:** Conceptualization, Funding acquisition, Project administration, Supervision, Validation, Writing – review & editing. **Thomas Haberer:** Conceptualization, Funding acquisition, Project administration, Supervision, Validation, Writing – original draft, Writing – review & editing. **Amir Abdollahi:** Conceptualization, Funding acquisition, Project administration, Supervision, Validation, Writing – review & editing. **Jürgen Debus:** Conceptualization, Funding acquisition, Project administration, Supervision, Validation, Writing – review & editing. **Thomas Tessonnier:** Data curation, Formal analysis, Investigation, Methodology, Resources, Software, Validation, Visualization, Supervision, Funding acquisition, Writing – original draft, Writing – review & editing. **Andrea Mairani:** Conceptualization, Data curation, Formal analysis, Investigation, Methodology, Resources, Software, Validation, Visualization, Project administration, Supervision, Funding acquisition, Writing – original draft, Writing – review & editing.

## Declaration of competing interest

The authors declare that they have no known competing financial interests or personal relationships that could have appeared to influence the work reported in this paper.
