# Supplementary material for: Mixed- and multi-relative biological effectiveness model simultaneous optimization in carbon ion radiotherapy: A proof-of-concept
Source: Phys Imaging Radiat Oncol. 2024 Nov 20;32:100679. doi: 10.1016/j.phro.2024.100679 (PMC11648266; doi:10.1016/j.phro.2024.100679)
Supplement: Supplementary Data 1 [file mmc1.docx]

**Supplementary material**

***Additional information on the case studies***


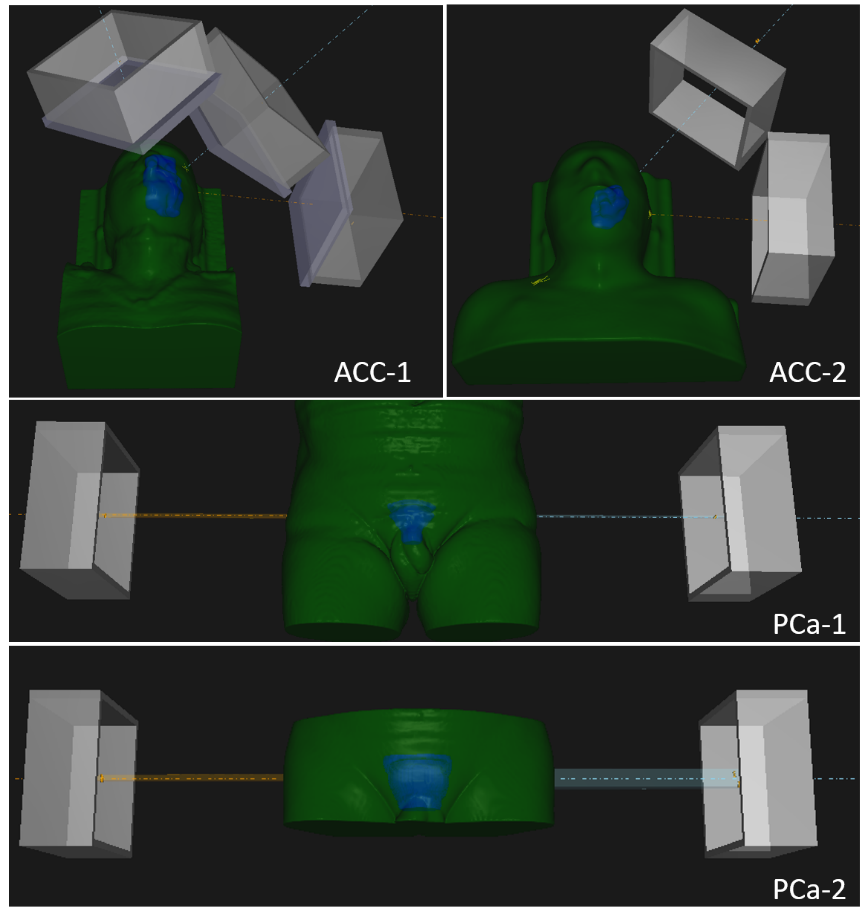
The beam parameters and spatial arrangement used in this study replicated the initial configuration employed for patient treatments at our institution. For ACC-1, the patient was treated at the isocentric gantry using three beams with range shifters. The gantry/table angles were 100°/0°, 50°/340°, and 340°/40°. The energy layer spacing was 3 mm in water, with a beam width in air at isocenter of approximately 10-11 mm and a hexagonal spot spacing of 3.6 mm. For ACC-2, the patient was treated at the horizontal beamline (90°). The table angles were 5° and 310°. The energy layer spacing was 3 mm in water, with a beam width in air at isocenter of approximately 6-8.5 mm and a hexagonal spot spacing of 2.4 mm. For PCa-1 and PCa-2, the patients were treated at the horizontal beamline (90°). The table angles were 0° and 180°. The energy layer spacing was 3 mm in water, with a beam width in air at isocenter of approximately 10 mm and a hexagonal spot spacing of 3.6 mm. The spatial arrangement for all four cases is depicted in Figure SM0.

**Figure SM0.** Beam configurations for the four patient cases.

**Table SM1**. Summary of the treatment plans’ features for the additional selected test cases: adenoid cystic carcinoma (ACC) for the head-and-neck region, and prostate cancer (PCa) for the pelvic region.

| *Case name-number* | *LEM-I Prescription dose* [Gy(RBE)_LEM-I_] | *mMKM/NIRS-MKM*  *Prescription dose* [Gy(RBE)_mMKM/JP_] | *LEM-I*  *Dose per fraction* [Gy(RBE)_LEM-I_] | *mMKM/NIRS-MKM*  *Dose per fraction* [Gy(RBE)_mMKM/JP_] | *Number of fractions* | *CTV size*  [cc] | *Fields’ arrangement* |
| --- | --- | --- | --- | --- | --- | --- | --- |
| ACC-2 | 51.0 | 51.0* | 3.00 | 3.00* | 17 | 93.3 | 2 ipsilateral beams |
| PCa-2 | 66.4 | 57.6** | 4.15 | 3.60** | 16 | 105.3 | 2 opposite beams |

* Gy(RBE)_mMKM_ (mMKM); ** Gy(RBE)_JP_ (NIRS-MKM)

**Table SM2**. Clinical goals for the additional selected test cases (ACC-2 and PCa-2) for each optimization scenario). Explicit numeric outcomes referring to the clinical target volume (CTV) are reported in terms of LEM-I and recalculated mMKM (ACC) or NIRS-MKM (PCa) biological doses. Additionally, the number of regions of interest (ROIs) achieving the desired dose objectives referring to the LEM-I for all organs at risk (OARs) is presented. The numerical findings are detailed in the referenced table of the supplementary material. A cross-mark denoting non-adherence (✕) to the dosimetric objectives for easier interpretation has been used.

| *Case name-number* | *ROI* | *Clinical goal* [Gy(RBE)_LEM-I_] | *ACC-2.A, PCa-2.A* | | *ACC-2.B, PCa-2.B* | | *ACC-2.C, PCa-2.C* | |
| --- | --- | --- | --- | --- | --- | --- | --- | --- |
|  |  |  | *LEM-I Value* [%] (*Result*) | *mMKM/NIRS-MKM Value* [%] (*Result*) | *LEM-I Value* [%] (*Result*) | *mMKM/NIRS-MKM Value* [%] (*Result*) | *LEM-I Value* [%] (*Result*) | *mMKM/NIRS-MKM Value* [%] (*Result*) |
| *ACC-2* | CTV | *V*_48.45_ ≥ 95.0% | 97.6 | 79.5 (✕) | 80.0 (✕) | 97.3 | 94.8 (✕) | 97.4 |
|  | OARs (8) | *(see* ***Table SM4*** *in* ***supplementary material****)* | all fulfilled | - | all fulfilled | - | all fulfilled | - |
| PCa-2 | CTV | *V*_54.72/63.08_* ≥ 95.0% | 100.0 | 100.0 | 98.9 | 100.0 | 100.0 | 100.0 |
|  | OARs (5) | *(see* ***Table SM6*** *in* ***supplementary material****)* | all fulfilled | - | all fulfilled | - | all fulfilled | - |

* *V*_LEM-I/NIRS-MKM_: subscripted dose values correspond to 95% of the prescription dose as according to the LEM-I and NIRS-MKM.

***Additional information for ACC-1 plan optimization***

**Table SM3**. LEM-I-derived dose objectives (clinical goals) for the ACC-1 case study evaluated for each optimization scenario in terms of LEM-I biological dose.

| *ACC-1* | | *ACC-1.A* | *ACC-1.B* | *ACC-1.C* |
| --- | --- | --- | --- | --- |
| *ROI* | *Clinical goal* [Gy(RBE)_LEM-I_] | *Value* [Gy(RBE)_LEM-I_] (*Result*) | *Value* [Gy(RBE)_LEM-I_] (*Result*) | *Value* [Gy(RBE)_LEM-I_] (*Result*) |
| Brainstem | *D*_0.03cc_ ≤ 36.0 | 35.7 | 35.1 | 35.5 |
| Optic nerve (right) | *D*_0.03cc_ ≤ 48.0 | 47.5 | 45.9 | 46.6 |
| Temporal lobe (left) | *D*_1cc_ ≤ 52.0 | 51.6 | 51.2 | 51.4 |
| Eye (left) | *D*_0.01cc_ ≤ 57.0 | 56.6 | 56.5 | 56.5 |
| Inner ear (left) | *D*_average_ ≤ 30.0 | 29.0 | 28.9 | 28.9 |
| Parotid (left) | *D*_average_ ≤ 23.0 | 22.6 | 22.3 | 22.4 |
| Lacrimal gland (left) | *D*_0.03cc_ ≤ 40.0 | 35.3 | 35.7 | 35.8 |
| Lacrimal gland (left) | *D*_average_ ≤ 23.0 | 21.4 | 21.3 | 21.5 |
| Optic chiasm | *D*_0.03cc_ ≤ 48.0 | 45.6 | 46.0 | 46.2 |
| Cochlea (left) | *D*_average_ ≤ 45.0 | 44.5 | 44.6 | 44.7 |
| Skin | *D*_0.03cc_ ≤ 54.0 | 53.0 | 53.5 | 52.9 |
| Lens (left) | *D*_average_ ≤ 5.0 | 3.5 | 3.6 | 3.7 |
| Lens (right) | *D*_average_ ≤ 5.0 | 2.5 | 3.1 | 3.0 |
| Mandible | *D*_0.03cc_ ≤ 57.0 | 56.3 | 56.5 | 56.5 |

***Additional information for ACC-2 plan optimization and results***

**
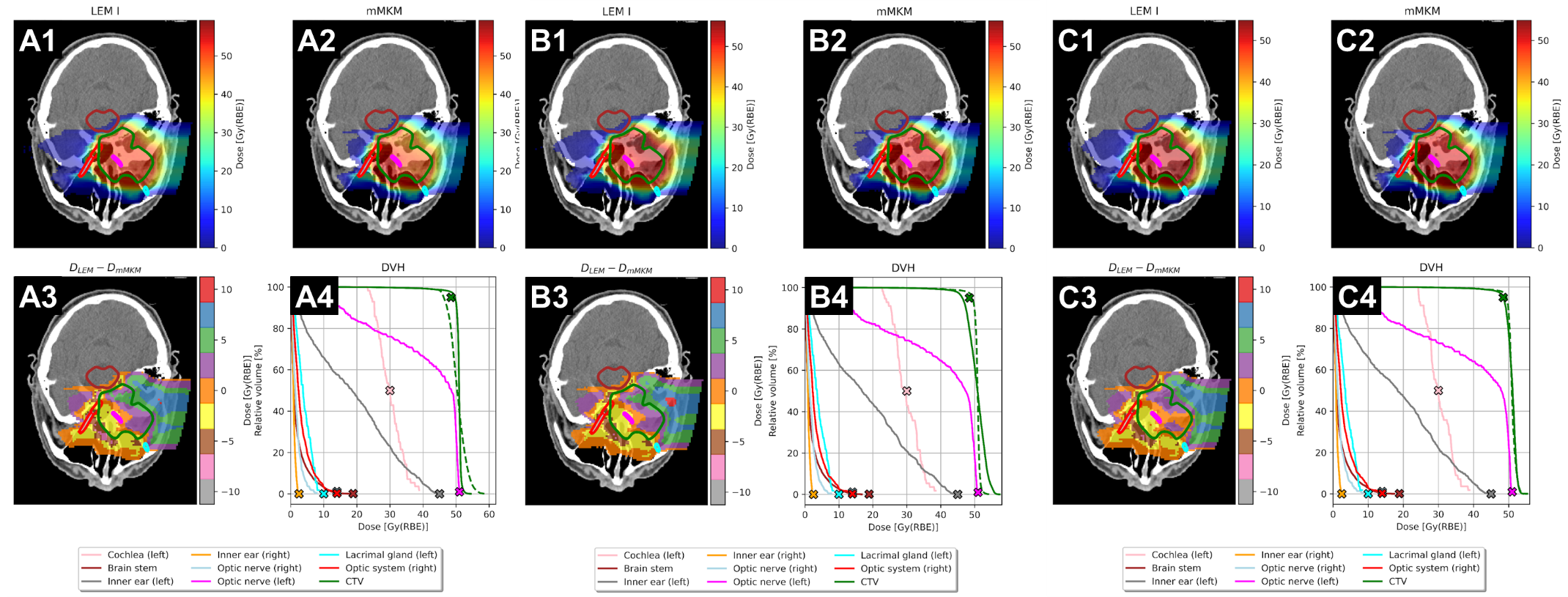
**

**Figure SM1.** ACC-2 case. Panels A) LEM-I-derived clinical goals for both OARs and targets; panels B) LEM-I-derived clinical goals for OARs and mMKM-derived clinical goals for targets; panels C) LEM-I-derived clinical goals for OARs and simultaneous LEM-I/mMKM-derived clinical goals for targets. Subfigures 1, 2, 3 and 4 depict resulting LEM-I, mMKM, dose difference distributions and DVHs. DVHs for LEM-I and mMKM are displayed as solid and dashed lines, respectively. The cross-marks represent the LEM-I dose objectives from Table SM3.

**Table SM4**. LEM-I-derived dose objectives (clinical goals) for the ACC-2 case study evaluated for each optimization scenario in terms of LEM-I biological dose.

| *ACC-2* | | *ACC-2.A* | *ACC-2.B* | *ACC-2.C* |
| --- | --- | --- | --- | --- |
| *ROI* | *Clinical goal* [Gy(RBE)_LEM-I_] | *Value* [Gy(RBE)_LEM-I_] (*Result*) | *Value* [Gy(RBE)_LEM-I_] (*Result*) | *Value* [Gy(RBE)_LEM-I_] (*Result*) |
| Brainstem | *D*_0.03cc_ ≤ 18.9 | 16.2 | 15.6 | 15.5 |
| Optic system (right) | *D*_0.01cc_ ≤ 14.0 | 13.7 | 13.9 | 13.5 |
| Optic nerve (left) | *D*_0.01cc_ ≤ 51.0 | 51.0 | 50.9 | 50.8 |
| Optic nerve (right) | *D*_0.01cc_ ≤ 14.0 | 7.0 | 7.8 | 7.4 |
| Inner ear (left) | *D*_max_ ≤ 45.0 | 45.0 | 45.0 | 45.0 |
| Inner ear (right) | *D*_max_ ≤ 2.5 | 2.5 | 2.2 | 2.4 |
| Lacrimal gland (left) | *D*_5%_ ≤ 10.0 | 7.7 | 7.7 | 7.2 |
| Cochlea (left) | *D*_50%_ ≤ 30.0 | 30.0 | 29.4 | 29.6 |

ACC-2.A. The same considerations as in the ACC-1.A apply here, for another dose prescription level. Qualitatively, the behavior of the LEM-I optimized dose distribution and its mMKM recalculation are similar to ACC-1.A, in terms of homogeneity (HI_LEM-I_ = 8.5% vs. HI_mMKM_ = 21.1 %), target coverage (D_50%_/D_98%_ values were 0.1%/6.3% and 1.4%/11.4% less than the prescribed dose for LEM-I and mMKM, respectively), hotspots (*D*_2cc_ reached 102.0% and 109.4%, for LEM-I and mMKM, respectively) and voxelwise dose difference.

ACC-2.B. The same trend in results observed for ACC-1.B are found for ACC-2.B, with respect to uniformity (HI_LEM-I_ = 21.4%, HI_mMKM_ = 9.0%), target coverage (*D*_98%_ values were 87.4% and 93.3% of the prescribed dose for LEM-I and mMKM, respectively), presence of hotspots (*D*_2cc_ values were 108.7% and 102.3% of the prescribed dose for LEM-I and mMKM, respectively) and dose differences, even though the prescription dose changed.

ACC-2.C. For this case, the dose distributions recalculated separately with the two models, LEM-I and mMKM, exhibited similarities when both radiobiological models were employed simultaneously to optimize the dose distribution on the targets. Similar to ACC-1.C, the dose objectives derived from LEM-I were achieved for all OARs and CTVs (fully with mMKM and violated by less than 0.2% for LEM-I). Median doses were within 0.3% of the prescribed dose level; *D*_2cc_ was around 104.2% for both models, and the most pronounced difference lied in coverage, as *D*_98%_ was 91.1% for the recalculated distribution in LEM-I, whereas it was 93.7% for mMKM. The disparity in CTV coverage was reflected in the HI of 12.9% (LEM-I) and 10.6% (mMKM), although no substantial discrepancy could be asserted. The dose difference map did not exhibit the same uniformity as in the ACC-1.C case.

***Additional information for PCa-1 plan optimization***

**Table SM5**. LEM-I-derived dose objectives (clinical goals) for the PCa-1 case study evaluated for each optimization scenario in terms of LEM-I biological dose.

| *PCa-1* | | *PCa-1.A* | *PCa-1.B* | *PCa-1.C* |
| --- | --- | --- | --- | --- |
| *ROI* | *Clinical goal* [Gy(RBE)_LEM-I_] | *Value* [Gy(RBE)_LEM-I_] (*Result*) | *Value* [Gy(RBE)_LEM-I_] (*Result*) | *Value* [Gy(RBE)_LEM-I_] (*Result*) |
| Bladder-PTV | *D*_0.03cc_ ≤ 66.0 | 65.9 | 64.9 | 66.0 |
| Bladder-PTV | *D*_50cc_ ≤ 50.0 | 25.2 | 26.3 | 26.1 |
| Rectum | *D*_1cc_ ≤ 66.0 | 64.2 | 63.5 | 65.8 |
| Rectum | *D*_5cc_ ≤ 61.0 | 56.1 | 56.3 | 58.5 |
| Rectum | *D*_10cc_ ≤ 54.0 | 46.2 | 46.8 | 48.1 |

***Additional information for PCa-2 plan optimization and results***

*
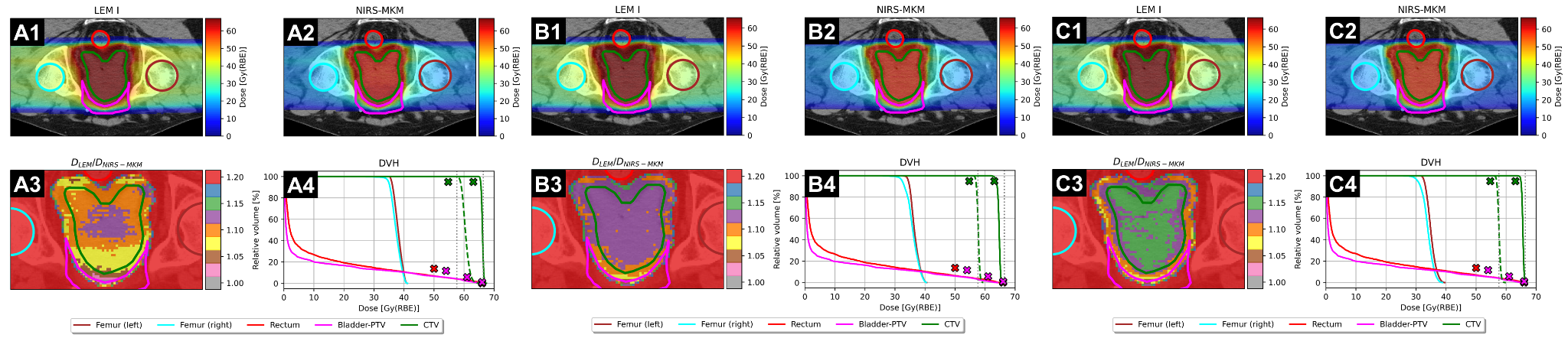
*

**Figure SM2.** PCa-2 case. Panels A) LEM-I-derived clinical goals for both OARs and targets; panels B) LEM-I-derived clinical goals for OARs and NIRS-MKM-derived clinical goals for targets; panels C) LEM-I-derived clinical goals for OARs and simultaneous LEM-I/NIRS-MKM-derived clinical goals for targets. Subfigures 1, 2, 3 and 4 depict resulting LEM-I, NIRS-MKM, dose ratio distributions and DVHs. DVHs for LEM-I and NIRS-MKM are displayed as solid and dashed lines, respectively. The cross-marks represent the LEM-I dose objectives from Table SM5. Vertical dotted lines in subfigures 4 represent nominal dose prescription at *D*_50%_, separately for the two models.

**Table SM6**. LEM-I-derived dose objectives (clinical goals) for the PCa-2 case study evaluated for the optimization scenarios 2 and 3 in terms of LEM-I biological dose.

| *PCa-2* | | *PCa-2.A* | *PCa-2.B* | *PCa-2.C* |
| --- | --- | --- | --- | --- |
| *ROI* | *Clinical goal* [Gy(RBE)_LEM-I_] | *Value* [Gy(RBE)_LEM-I_] (*Result*) | *Value* [Gy(RBE)_LEM-I_] (*Result*) | *Value* [Gy(RBE)_LEM-I_] (*Result*) |
| Bladder-PTV | *D*_0.03cc_ ≤ 66.0 | 66.0 | 64.3 | 65.3 |
| Bladder-PTV | *D*_50cc_ ≤ 50.0 | 26.6 | 25.5 | 26.5 |
| Rectum | *D*_1cc_ ≤ 66.0 | 64.8 | 63.1 | 64.3 |
| Rectum | *D*_5cc_ ≤ 61.0 | 52.6 | 51.5 | 52.9 |
| Rectum | *D*_10cc_ ≤ 54.0 | 37.6 | 37.0 | 37.8 |

PCa-2.A. The same considerations as those outlined in PCa-2.A are applicable here. The performance of the LEM-I optimized dose distribution, along with its NIRS-MKM recalculation, mirrored previous findings regarding homogeneity (HI_LEM-I_ = 2.3% vs. HI_NIRS-MKM_ = 7.9 %) and hotspots (*D*_2cc_ reached 100.7% and 109.9%, for LEM-I and NIRS-MKM, respectively). The ratio between median doses of the CTV when optimizing in LEM-I and recalculating in NIRS-MKM was 1.095, which is below the theoretical prediction (1.15) by Fossati et al.[1] and Molinelli et al.[2].

PCa-2.B. Analogous considerations to those in PCa-1.B are exhibited. Both dose distributions demonstrated similar HI (HI_LEM-I_ = 3.3%, HI_NIRS-MKM_ = 3.4%). Notably, the LEM-I distribution consistently displayed a dose underestimation compared to the rescaled value predicted by the theoretical model, with the CTV *D*_50%_ registering 2.8% lower than LEM-I prescription dose level. In contrast, the dose distribution in NIRS-MKM achieved desired dose level and homogeneity within the target (*D*_50%_ = 100.2%, *D*_98%_ = 98.5% and *D*_2cc_ = 101.9% of the NIRS-MKM prescription dose). The resulting *F*_scal_ was 1.12.

PCa-2.C. For this prostate case, while uniformly distributed (HI_LEM-I_ = 2.3%) without hotspots, with target coverage *V*_95%_ objective achieved, the LEM-I recalculation revealed a slight underdosage of the CTV median doses by 1.2%, compared to the prescribed value. The NIRS-MKM distribution showed a high-level dose homogeneity (HI_NIRS-MKM_ = 3.7%) and matched the prescription dose, with CTV median dose deviating by less than 0.1%. Compared to PCa-1.C, the scaling coefficient was slightly underestimated by approximately 1.2% relative to the expected value.

***Additional information on full Monte Carlo treatment planning system based on MonteRay***

The newly implemented fluence optimization module identified the optimal vector of scan spot weights $x\in{R^{n}}_{\geq0}$ by solving the following optimization problem:

| $\underset{x \in\Omega}{\min C\left( x \right)}:=\sum_{i = 1}^{N} w_{i}C_{i}\left( x \right) , \Omega=\left\{ x\in{R^{n}}_{\geq0} \vert g_{k}\left( x \right)\leq0, k=1, ... , M \right\}$ | (1) |
| --- | --- |

where the cost function *C* to be minimized is defined as the weighted sum of individual cost function terms *C_i_*, and *g_k_* represents optional supplementary constraints for the feasible solution set. The non-negativity condition $x\geq0$ ensures that the resultant vector of scan spots weights excludes negative values, as negative numbers are infeasible for delivery.

Each cost function term and optional constraint assesses the dose distribution within its respective region of interest (ROI) based on a specific metric. These functions yield higher values for dose distributions that deviate more strongly from their corresponding dose goals. For example, they may compute the sum of squared differences between the dose values in a given ROI and a specified target dose. The implementation of cost function terms and constraints are largely, but not exclusively, congruent with those available in research and/or commercial TPSs. The optimization module accommodates a diverse array of cost function term types, largely consistent with those outlined in matRad [3], including minimum/maximum/target dose, mean dose, equivalent uniform dose (EUD), and minimum/maximum dose-volume histogram (DVH) cost functions. Minimum and maximum cost function terms penalize deviations from the target values in either the positive or the negative direction, while target cost function terms consider deviations in both directions. Moreover, all unidirectional types of cost function terms can alternatively function as constraints.

The optimization process operates on multiple GPUs in parallel, which significantly reduces optimization time and addresses GPU memory limitations. Existing fluence optimizers are either not (multi-)GPU-based[3], potentially limiting optimization performance, or they are not designed for particle therapy plan optimization[4,5], offering a very limited set of cost function and constraint types.

The fluence optimizer offers the flexibility to consider both physical dose and biological dose, incorporating models like LEM-I, mMKM, NIRS-MKM, and constant RBE. Each cost function term or constraint allows for the individual selection of dose type, enabling optimization across multiple RBE models concurrently. The biological dose *D*_RBE_ for a single voxel in different models can be computed using the provided formula (voxel index omitted for readability):

| $D_{\mathrm{RBE}}=-\left( \frac{\alpha_{r}}{{2\beta}_{r}} \right)+\sqrt{\left( \frac{\alpha_{r}}{{2\beta}_{r}} \right)^{2}+\frac{\bar{\alpha}d_{\mathrm{PHY}}+\left( \sqrt{\bar{\beta}}d_{\mathrm{PHY}} \right)^{2}}{\beta_{r}}}$ | (2) |
| --- | --- |

where $\alpha_{r}$ and $\beta_{r}$ represent the linear-quadratic (LQ) model parameters for the reference (*r*) radiation, while *d*_PHY_ represents the physical dose, $\bar{\alpha}$ and $\sqrt{\bar{\beta}}$ denote the dose-weighted average LQ parameters for the selected radiobiological model. The weighted average is calculated over the dose contribution of each individual particle in the mixed radiation field, using energy-dependent LQ parameters, according to the Zaider and Rossi’s formalism based on the theory of dual radiation action [6], namely:

| $\bar{\alpha}_{j}=\frac{\sum_{i} d_{ij}\alpha_{ij}}{\sum_{i} d_{ij}} , {\sqrt{\bar{\beta}}}_{j}=\frac{\sum_{i} d_{ij}\sqrt{\beta}_{ij}}{\sum_{i} d_{ij}}$ | (3) |
| --- | --- |

where $d_{ij}$ is the dose from the *i*^th^ particle with associated pre-calculated $\alpha_{ij}$, $\sqrt{\beta}_{ij}$ in voxel *j*, and *i* runs over all particles depositing dose in voxel *j* [7,8].

Specifically, for the mMKM and the mMKM-derived NIRS-MKM, due to the linear relationship between $\bar{\alpha}$ and the saturation-corrected dose-mean specific energy $\bar{z}_{1D}^{*}$, as shown in the following equation,

| $\bar{\alpha}=\alpha_{0}+\beta_{r}\bar{z}_{1D}^{*} , \sqrt{\bar{\beta}}\equiv\sqrt{\beta_{r}}$ | (4) |
| --- | --- |

with $\alpha_{0}$ being the initial slope of the survival curve in the limit of vanishing LET, equation (3) simplifies to only scoring the dose-weighted $\bar{z}_{1D}^{*}$:

| $\bar{z}_{1Dj}^{*}=\frac{\sum_{i} d_{ij}z_{1Dij}^{*}}{\sum_{i} d_{ij}}$ | (5) |
| --- | --- |

Equation 2 needs to be rescaled by a constant factor *F*_clin_ = 2.41 to obtain the biological dose for the NIRS-MKM [9].

Table SM7 lists the input parameters for the above-mentioned models. For both LEM-I and mMKM, the values of $\alpha_{0}$, $\alpha_{r}$ and $\beta_{r}$ used are those implemented in our treatment planning system and clinical workflow for treating patients with helium and, more recently, with carbon ions for specific clinical indications . While the specific choice of $\alpha_{r}$ and $\beta_{r}$ is irrelevant for LEM-I, as the model depends solely on their ratio, the mMKM explicitly depends on the value of $\beta_{r}$. The approximation $\alpha_{0}=\alpha_{r}$ is commonly employed, either implicitly or explicitly, in various phenomenological and mechanistic biological models. The mMKM implementation has been benchmarked against in vitro and in vivo data in the literature for proton, helium, and carbon ions [10,11]. Additionally, initial validation with clinical data for proton and carbon ions further supports the soundness of this approximation [12]. The NIRS-MKM parameters were obtained from the published reference [9].

**Table SM7**. Input parameters for the radiobiological models handled by the MonteRay optimization module.

| RBE model | $\alpha_{0}$[Gy^-1^] | $\alpha_{r}$[Gy^-1^] | $\beta_{r}$[Gy^-2^] |
| --- | --- | --- | --- |
| LEM-I | - | 0.1 | 0.05 |
| mMKM * | 0.05 | 0.05 | 0.025 |
| NIRS-MKM ** | 0.172 | 0.764 | 0.0615 |

**R_d_* = 0.30 µm, *R_n_* = 3.6 µm [11,13]; ***R_d_* = 0.32 µm, *R_n_* = 3.9 µm [14], with *R_d_* and *R_n_* being the radius of the domain and of the nucleus, respectively.

It is worth noticing that for LEM-I and mMKM input tables were generated using the same $\left( \alpha/\beta\right)_{r}$​ ratio value across the two models. This approach ensures a direct dosimetric comparison between the two models by standardizing the reference radiation conditions.

The optimizer interfaces with the MC dose engine MonteRay [15–17] to generate dose influence data essential for the optimization process. This data includes dose influence matrices (such as dose-weighted $\alpha$ and $\sqrt{\beta}$ matrices, etc., generically known as dose influence data) for all ROIs and biological models involved in defining the optimization problem. The dose influence data is obtained through separate MC simulations by iterating over all initial scan spots and simulating *N_p_* = 5000 primaries [18]. For each ROI considered in the optimization, a binary mask is generated using a scanline algorithm. These masks are then used to store the respective influence data for all voxels within the ROIs as sparse matrices.

Furthermore, while only LEM-I, NIRS-MKM and mMKM are presented here, any models could also be implemented within this framework. This flexibility could even accommodate future developments in proton therapy, such as a potential transition from a constant to a variable RBE description.

To tackle the fluence optimization problem, the optimizer relies on IPOPT, a publicly available framework tailored for large-scale nonlinear optimization[19]. IPOPT's optimization procedure requires multiple evaluations of the cost function, constraints, and their gradients to generate a series of steps converging towards a (local) minimizer of the fluence optimization problem.

Given the computationally demanding nature of this task, which is nevertheless highly susceptible to parallelization, multiple graphics processing units (GPUs) are employed simultaneously. This is achieved by storing the dose influence data for each ROI and type of dose onto separate GPUs.

During each iteration, every GPU is tasked with evaluating the pertinent cost function terms and constraints, utilizing the dose influence data stored within its memory. This methodology facilitates the parallel evaluation of multiple cost function terms and constraints through GPU acceleration. Subsequently, the individual cost function terms and their gradients are summed up to derive the total cost function's gradient and function value. This information, along with constraint values and their gradients, is forwarded to IPOPT for the computation of the subsequent iteration.

This iterative process is repeated until IPOPT's convergence criteria, which can be adjusted by the user, are met, ultimately yielding the solution that is then returned to the user.

***Supplementary references***

[1] Fossati P, Molinelli S, Matsufuji N, Ciocca M, Mirandola A, Mairani A, et al. Dose prescription in carbon ion radiotherapy: A planning study to compare NIRS and LEM approaches with a clinically-oriented strategy. Phys Med Biol 2012;57:7543–54. https://doi.org/10.1088/0031-9155/57/22/7543.

[2] Molinelli S, Magro G, Mairani A, Matsufuji N, Kanematsu N, Inaniwa T, et al. Dose prescription in carbon ion radiotherapy: How to compare two different RBE-weighted dose calculation systems. Radiother Oncol 2016;120. https://doi.org/10.1016/j.radonc.2016.05.031.

[3] Wieser HP, Cisternas E, Wahl N, Ulrich S, Stadler A, Mescher H, et al. Development of the open-source dose calculation and optimization toolkit matRad. Med Phys 2017;44:2556–68. https://doi.org/10.1002/MP.12251.

[4] Tian Z, Peng F, Folkerts M, Tan J, Jia X, Jiang SB. Multi-GPU implementation of a VMAT treatment plan optimization algorithm. Med Phys 2015;42. https://doi.org/10.1118/1.4919742.

[5] Men C, Gu X, Choi D, Majumdar A, Zheng Z, Mueller K, et al. GPU-based ultrafast IMRT plan optimization. Phys Med Biol 2009;54:6565–73. https://doi.org/10.1088/0031-9155/54/21/008.

[6] Zaider M, Rossi HH. The synergistic effects of different radiations. Radiat Res Soc 1980;83:732–9.

[7] Mairani A, Brons S, Cerutti F, Fassò A, Ferrari A, Krämer M, et al. The FLUKA Monte Carlo code coupled with the local effect model for biological calculations in carbon ion therapy. Phys Med Biol 2010;55:4273–89. https://doi.org/10.1088/0031-9155/55/15/006.

[8] Magro G, Dahle TJ, Molinelli S, Ciocca M, Fossati P, Ferrari A, et al. The FLUKA Monte Carlo code coupled with the NIRS approach for clinical dose calculations in carbon ion therapy. Phys Med Biol 2017;62:3814–27. https://doi.org/10.1088/1361-6560/aa642b.

[9] Inaniwa T, Kanematsu N, Matsufuji N, Kanai T, Shirai T, Noda K, et al. Reformulation of a clinical-dose system for carbon-ion radiotherapy treatment planning at the National Institute of Radiological Sciences, Japan. Phys Med Biol 2015;60:3271–86. https://doi.org/10.1088/0031-9155/60/8/3271.

[10] Mein S, Dokic I, Klein C, Tessonnier T, Böhlen TTTTTTTT, Magro GG, et al. Biophysical modeling and experimental validation of relative biological effectiveness (RBE) for 4He ion beam therapy. Radiat Oncol 2019;14:1–16. https://doi.org/10.1186/s13014-019-1295-z.

[11] Mein S, Klein C, Kopp B, Magro G, Harrabi S, Karger CP, et al. Assessment of RBE-Weighted Dose Models for Carbon Ion Therapy Toward Modernization of Clinical Practice at HIT: In Vitro, in Vivo, and in Patients. Int J Radiat Oncol Biol Phys 2020;108:779–91. https://doi.org/10.1016/j.ijrobp.2020.05.041.

[12] Besuglow J, Tessonnier T, Mein S, Eichkorn T, Haberer T, Herfarth K, et al. Understanding RBE and clinical outcome of prostate cancer therapy using particle irradiation: analysis of tumor control probability with mMKM. Int J Radiat Oncol 2024;000:1–12. https://doi.org/10.1016/j.ijrobp.2024.02.025.

[13] Mairani A, Magro G, Tessonnier T, Böhlen TTT, Molinelli S, Ferrari A, et al. Optimizing the modified microdosimetric kinetic model input parameters for proton and 4He ion beam therapy application. Phys Med Biol 2017;62:N244–56. https://doi.org/10.1088/1361-6560/aa6be9.

[14] Inaniwa T, Furukawa T, Kase Y, Matsufuji N, Toshito T, Matsumoto Y, et al. Treatment planning for a scanned carbon beam with a modified microdosimetric kinetic model. Phys Med Biol 2010;55:6721–37. https://doi.org/10.1088/0031-9155/55/22/008.

[15] Lysakovski P, Ferrari A, Tesonnier T, Besuglow J, Kopp B, Mein S, et al. Development and Benchmarking of a Monte Carlo Dose Engine for Proton Radiation Therapy. Front Phys 2021;9:1–14. https://doi.org/10.3389/fphy.2021.741453.

[16] Lysakovski P, Kopp B, Tessonnier T, Mein S, Ferrari A, Haberer T, et al. Development and validation of MonteRay, a fast Monte Carlo dose engine for carbon ion beam radiotherapy. Med Phys 2023:1–17. https://doi.org/10.1002/mp.16754.

[17] Lysakovski P, Kopp B, Tessonnier T, Mein S, Ferrari A, Haberer T, et al. Development and validation of MonteRay, a fast Monte Carlo dose engine for carbon ion beam radiotherapy. Med Phys 2024;51:1433–49. https://doi.org/10.1002/MP.16754.

[18] Mairani A, Böhlen TT, Schiavi A, Tessonnier T, Molinelli S, Brons S, et al. A Monte Carlo-based treatment planning tool for proton therapy. Phys Med Biol 2013;58:2471–90. https://doi.org/10.1088/0031-9155/58/8/2471.

[19] Wächter A, Biegler LT. On the implementation of an interior-point filter line-search algorithm for large-scale nonlinear programming. Math Program 2006;106:25–57. https://doi.org/10.1007/S10107-004-0559-Y/METRICS.
